# Do multiple personal roles promote working energetically in female nurses? A cross-sectional study of relevant factors promoting work engagement in female nurses

**DOI:** 10.1186/s12199-019-0810-z

**Published:** 2019-09-12

**Authors:** Nagisa Okada, Kosuke Yabase, Toshio Kobayashi, Hitoshi Okamura

**Affiliations:** 1 Nursing Science and Arts, School of Health Sciences, University of Occupational and Environmental Health, 1-1 Iseigaoka, Yahatanishi-ku, Kitakyushu City, Fukuoka 807-8555 Japan; 2 Graduate School of Biomedical and Health Sciences, Hiroshima University, 1-2-3 Kasumi, Minami-ku, Hiroshima City, Hiroshima, 734-8551 Japan; 3 Ishii Memorial Hospital, 3-102-1 Tada, Iwakuni City, Yamaguchi 741-8585 Japan

**Keywords:** Work engagement, Spillover, Multiple roles, Female nurses, Coping

## Abstract

**Background:**

Like most women, female nurses in the workforce experience life events such as marriage, childbirth, and child-rearing, and carry out numerous personal roles. This may result in an increase in various demands for nurses, and coping with these roles may promote work engagement. However, few studies have focused on work engagement or spillover effects, including those in the family domain, in female nurses with multiple roles. In the present study, we aimed to examine work engagement in female nurses and investigate its relationship with factors such as the presence or absence of multiple personal roles.

**Methods:**

The subjects of this study were 1225 female nurses working at three general hospitals, each with at least 200 hospital beds in Fukuoka Prefecture, Japan. The cross-sectional design of the study used anonymous self-administered questionnaires. Responses were received from 650 nurses (response rate 53.1%), of which 612 were valid (valid response rate 50.0%). Multiple regression analysis was performed on the 612 responses regarding associations between work engagement and the presence or absence of multiple roles (role as a wife or mother), spillover effects, coping characteristics, job demands, and job resources.

**Results:**

In general, the work engagement of female nurses was low, as is the case with other female workers in Japan, but work engagement was higher among female nurses with multiple roles than among those without. The regression analysis showed that factors associated with better work engagement in female nurses were family-to-work positive spillover, job resources, coping strategies including “changing a point of view,” “active solution for problems,” “avoidance and suppression,” and the presence of multiple roles.

**Conclusions:**

The results indicate that in addition to resources in the work domain, a family-to-work positive spillover effect, which is a variable in the non-work domain, may also promote energetic work among female nurses. Therefore, it is necessary for nurses to receive support at work and use effective coping strategies.

## Background

In recent years, various initiatives have been undertaken to improve workers’ mental health. Moreover, the conventional concept of salutogenesis has been developed to complement pathogenesis [1] and mental health assessment of workers is increasingly focusing on positive aspects such as well-being. Interest is particularly increasing in work engagement, a term describing a positive state of mind with respect to character strengths and work, proposed by Schaufeli et al. [2, 3]. Work engagement is defined as “a positive, fulfilling, work-related state of mind that is characterized by vigor, dedication, and absorption.” [2]. The job demands-resources (JD-R) model explains that work engagement comprises two processes: (1) a health impairment process, which explains the relationship between health problems and stress responses, with the level of work demand as a determinant; and (2) a motivational process, which explains work engagement and positive actions with work resources as a determinant [3, 4]. Previous studies on workers have found advanced age [5, 6] and low work-related stress [7, 8] were determinants of improved work engagement; further, the research has suggested that work engagement outcomes include improved mental health and better work performance of individuals and organizations [4, 9–18]. Previous studies also suggest that improving workers’ mental health, so that they can work energetically, may be important in turn for improving the mental health and performance of the workers themselves.

According to the National Institute for Occupational Safety and Health occupational stress model that explains the mental and physical health of workers, adding “non-work factors” such as domestic and family demands to “job stressors” impairs mental and physical health [19]. In particular, nursing involves many job stressors due to the tremendous responsibility of carrying out work related to the life and death of patients, the demand for emotional labor, and the need for shift work. Moreover, female nurses also have family roles outside of their job roles that involve life events such as marriage, childbirth, and child-rearing. These roles are reportedly associated with non-work factors and may lead to poorer mental and physical health [20]. In nurses with experiences such as marriage, childbirth, and childcare, “depersonalization” of burnout is higher than that in men, and changes in the family environment due to these life events might be a factor [21]. Moreover, in Japan, the primary reasons for full-time nurses quitting include marriage, childbirth, and child-rearing [22]. Further, despite 77.6% of potential nurses wishing to return to work, the primary reasons for not doing so included “child-rearing” and “difficulty achieving a work-family balance”; this has not changed in about 20 years [23, 24]. Therefore, in this study, we focused on female nurses.

On the other hand, studies on work engagement and burnout have shown that nurses with multiple roles, who are married, have a higher level of work engagement than those who are not married [25] and that nurses with children have a higher sense of achievement regarding work and are less likely to experience burnout [26]. These results suggest that having multiple roles may promote energetic working in female nurses. However, the JD-R model only explains the occurrence of health impairments and positive outcomes in job performance in terms of work-related variables such as “job demands,” “job resources,” and “work engagement,” aside from personal resources and stress responses [3, 4]. To examine the work-life of female nurses who experience life events such as marriage, childbirth, and child-rearing, it is necessary to consider not only work-related variables but also family-related ones. For variables that are related to both domains, there is a spillover effect between work and family; this means that any event or situation arising in a role in either the “work domain” or the “family domain” affects circumstances of the roles in the other domain [27]. From the two qualities (positive and negative) and two directions (work-to-family and family-to-work), there are four types of spillover: work-to-family negative spillover (WFNS), family-to-work negative spillover (FWNS), work-to-family positive spillover (WFPS), and family-to-work positive spillover (FWPS) [28]. According to past studies, if having multiple roles are perceived to be difficult, negative spillover such as WFNS or FWNS leads to negative outcomes, such as psychological distress [28, 29]. In contrast, the presence of multiple roles can also have positive outcomes, and positive spillover may promote better health (mental and physical), a higher level of satisfaction (with work and family), and better performance [30–33].

Based on the studies above, we can predict that, for female nurses, carrying out numerous roles may result in an increase in various demands, and coping with all those roles may be a strength in work life. However, few studies have focused on work engagement or spillover effects after including the family domain in female nurses with multiple roles. By identifying factors that promote working energetically, including having multiple roles, female nurses will be able to perceive life events such as marriage, childbirth, and child-rearing positively that earlier inhibited them to work and work energetically maintaining good mental and physical health.

Therefore, the study hypothesizes that

H1: The work engagement of female nurses is higher among those with multiple roles than among those without multiple roles.

H2: The factors associated with work engagement in female nurses are job resources, high WFPS and FWPS, low WFNS and FWNS, and the presence of multiple roles.

## Method

### Aim

We aimed to clarify the current state of work engagement in female nurses and its relationship with factors, such as the presence of multiple roles.

### Participants and procedure

Subjects were 1225 female nurses working at three general hospitals, each with at least 200 hospital beds in Fukuoka Prefecture, Japan. The study was a cross-sectional design using anonymous self-administered questionnaires. A total of 1225 questionnaires were distributed and 650 responses were received (response rate 53.1%). Of those, 612 were valid (valid response rate 50.0%). The 612 subjects who provided valid responses were divided into two groups based on the presence or absence of multiple roles. Multiple roles in this study were defined as work roles and roles played as a result of life events such as marriage, childbirth, and child-rearing. The main roles refer to worker roles and wife or mother roles, as life events such as marriage, childbirth, and child-rearing in Japan can lead to women discontinuing their nursing profession. As a result, 262 nurses had multiple roles and 350 did not. We calculated the power based on previous studies and confirmed that the minimum sample size needs to be at least 100. In this study, the number of female nurses with multiple roles was 262, indicating an appropriate sample size.

While distributing and collecting the questionnaires, subjects were provided with a written explanation about the purpose and methods of the study and were assured that participation was voluntary. Submission of a completed questionnaire was considered to indicate consent. Questionnaires were returned via post.

The entire procedure followed in the study was reviewed and approved by the Ethics Committee of the University of Occupational Environmental and Health Japan (Approval No. H28-156).

### Measures

Work engagement was assessed using the short-form Japanese version of the Utrecht Work Engagement Scale (UWES-J) created by Shimazu et al. based on the simplified version of the original UWES created by Schaufeli and Bakker [16, 34]. The short-form UWES-J has three subscales—“vigor,” “dedication,” and “absorption”—and comprises nine items on a 7-point scale. Higher scores correspond to stronger work engagement. The reliability and validity of the UWES-J have been demonstrated in previous studies (Cronbach’s alpha = 0.92) [35–37]; in the current study, the Cronbach’s alpha was 0.94.

Job demands and job resources were assessed with items from the new Brief Job Stress Questionnaire [38]. The job demands section comprised 13 items on topics such as quantitative and qualitative workload and role conflict. The job resources section comprised 27 items on topics such as job resources and support from bosses and colleagues. Items in both sections were assessed on a 4-point scale with possible responses of “strongly agree,” “agree,” “disagree,” and “strongly disagree.” Higher scores corresponded to more job demands and more job resources, respectively. The reliability and validity of the new Brief Job Stress Questionnaire have been demonstrated in a previous study (Cronbach’s alpha > 0.70) [39]; in the current study, the Cronbach’s alpha was 0.81.

Spillover effects were measured using the Japanese version of the Survey Work–Home Interaction-NijmeGen (SWING) created by Geurts et al. [40] and translated by Shimada, Shimazu, Kawakami, et al. [41] The Japanese version of the SWING (SWING-J) has four subscales (WFNS, FWNS, WFPS, and FWPS) comprising 22 items measured on a 4-point scale with possible responses of “never,” “sometimes,” “often,” and “always.” Higher scores correspond to more spillover in each scale, respectively. This scale measures the balance between “work” and “home,” which includes spouse, family members, and friends. The reliability and validity of the SWING-J have been demonstrated in a previous study (Cronbach’s alpha = 0.75–0.86) [41]; in the current study, the Cronbach’s alpha was 0.83.

Characteristics of coping, which is a personal resource, were assessed with the Brief Scales for Coping Profile (BSCP) for workers created by Kageyama et al. [42–44] The BSCP has six subscales: “active solution for problems” and “seeking help for solution” as problem-focused coping and “changing mood,” “changing a point of view,” “emotional expression involving others,” and “avoidance and suppression” as emotion-focused coping. It comprises 18 questions measured on a 4-point scale with possible responses of “rarely,” “occasionally,” “sometimes,” and “often.” Higher scores corresponded to more frequent use of that type of coping. The reliability and validity of BSCP have been demonstrated in previous studies (Cronbach’s alpha = 0.66–0.79) [43–45]; in the current study, the Cronbach’s alpha was 0.77.

Attributes investigated were age, years of experience, frequency of night shifts per month, position, type of employment, department, shift format, marital status, presence or absence of a spouse, cohabitation status with spouse, employment of spouse, presence or absence of children, number of children, presence or absence of children under school age, and the number of children under school age.

### Data analyses

Descriptive statistics were calculated for attributes and scores for each scale. To compare the two groups (presence and absence of multiple roles), Fisher’s exact tests and chi-square tests were performed on the subjects with each attribute, and Mann–Whitney *U* tests on the scores of each scale. For all subjects, simple correlation tests were performed with work engagement as the dependent variable and each measure as an independent variable. Next, hierarchical multiple regression analysis was performed on variables found to be significantly associated with work engagement in simple correlation tests (*p* < 0.05) and variables found to differ between groups (*p* < 0.05) with years of experience and presence or absence of multiple roles as independent variables. The Kolmogorov–Smirnov test was used to assess the normality of work engagement as a dependent variable. If the data were not normally distributed, the distribution was corrected with Blom’s normal score transformation. Variables were included by forced entry for each category, and a dummy variable was included for the presence or absence of multiple roles (0, without multiple roles; 1, with multiple roles). Multicollinearity between variables was verified with a criterion of variance inflation factor < 2. The analysis was performed using SPSS Statistics version 24 statistical software (IBM).

## Results

In this study, participants were recruited from three general hospitals in Fukuoka Prefecture, Japan between January and March 2017. Since nursing services can differ with the number of beds and functions of a hospital, we included hospitals with 200 beds or more to eliminate any bias.

Responses were obtained from 650 nurses (response rate 53.1%), of which responses from 38 participants were discarded due to missing data. Of the 612 valid responses (valid response rate, 50.0%), 262 nurses had multiple roles (married with no children; married with children; unmarried, divorced, or widowed with children), and 350 nurses did not have multiple roles (unmarried; divorced with no children).

Table 1 displays the subject attributes in the study. Subjects with multiple roles were significantly older and had significantly more experience than those without multiple roles. Regarding the frequency of night shifts per month, type of employment, department, and shift format, a higher proportion of subjects with multiple roles were part-time employees (15.6%), worked in the outpatient department (43.1%), and worked during the daytime hours (48.5%), while a higher proportion of subjects without multiple roles were regular member of staff (98.8%), worked in a hospital ward (76.0%), and worked in a two-shift system (80.0%).

**Table 1:** Subject Attributes

Item	All subjects (*N* = 612)			Subjects with multiple roles (*N* = 262)					Subjects without multiple roles(*N* = 350)					*p*
	Median	Range		Median	Range		Percentile		Median	Range		Percentile		
		Min	Max		Min	Max	25	75		Min	Max	25	75	
Age (years)	34.0	22	63	39.5	24	63	33.0	46.0	28.0	22	59	25.0	38.0	< 0.001 ^a^
Years of experience (years)	12.0	1	42	15.0	1	42	9.8	21.0	5.0	1	37	3.0	14.0	< 0.001 ^a^
Frequency of night shifts per month (time)	4.0	0	8	1.0	0	7	0.0	4.0	5.0	0	8	3.8	5.0	< 0.001 ^a^
		*N*	%				*N*	%				*N*	%	
Position														
Principal		24	3.9				13	5.0				11	3.1	0.351^b^
Chief		46	7.5				23	8.8				23	6.6	
Staff		539	88.1				224	85.5				315	90.0	
Other		3	0.5				2	0.8				1	0.3	
Type of employment														
Regular member of staff		567	92.6				221	84.4				346	98.9	< 0.001^b^
Non-regular member of staff		45	7.4				41	15.6				4	1.1	
Department														
Outpatient department		157	25.7				113	43.1				44	12.6	< 0.001^c^
Ward		398	65.0				132	50.4				266	76.0	
Other		57	9.3				17	6.5				40	11.4	
Shift format														
Day shift		177	28.9				127	48.5				50	14.3	< 0.001^b^
Three-shift system		4	0.7				0	0.0				4	1.1	
Two-shift system		398	65.0				118	45.0				280	80.0	
Other		33	5.4				17	6.5				16	4.6	
Marital status														
Unmarried		351	57.4				1	0.4				350	100.0	—
Married		227	37.1				227	86.6				0	0.0	
Divorced		29	4.7				29	11.1				0	0.0	
Bereavement		5	0.8				5	1.9				0	0.0	
Presence or absence of a spouse														
Presence of a spouse		—	—				227	86.6				—	—	—
Absence of a spouse		—	—				35	13.4				—	—	—
Cohabitation status with spouse (*N* = 227)														
Living together		—	—				212	93.4				—	—	—
Living separately		—	—				15	6.6				—	—	—
Employment of spouse (*N* = 227)														
At work		—	—				224	98.7				—	—	—
Out of work		—	—				3	1.3				—	—	—
Presence or absence of children														
Presence of children		—	—				196	74.8				—	—	—
Absence of children		—	—				66	25.2				—	—	—
Presence or absence of children under school age (*N* = 196)														
Presence of children under school age		―	—				72	36.7				—	—	—
Absence of children under school age		―	—				124	63.3				—	—	—
Number of children				2.0	1	7	1.0	2.0						
Number of children under school age				1.0	0	3	1.0	2.0						

^a^Comparison of subjects with and without multiple roles using Mann-Whitney *U* test

^b^Comparison of subjects with and without multiple roles using Fisher’s exact test

^c^Comparison of subjects with and without multiple roles using chi-square test

Table 2 displays the overall comparisons for coping, job demands, job resources, spillover, and work engagement variables based on the presence or absence of multiple roles. The BSCP score, which is a personal resource, revealed significant group differences in three of the six coping strategies; moreover, the scores for “active solution for problems” were higher among those with multiple roles than those without (*p* < 0.001). Meanwhile, scores for the strategy of “changing mood” were lower among those with multiple roles than those without (*p* = 0.002) and scores for “avoidance and suppression” were also lower among those with multiple roles than those without (*p* < 0.001). For the variables of “job demands” and “job resources” that assess the work domain, there was only a significant difference between groups in the former, with higher scores observed among nurses without multiple roles than nurses with multiple roles (*p* = 0.001). For spillover effects, which assessed the work-life domain, significant differences were observed between groups in three of the four subscales and scores for WFNS, a type of negative spillover, were higher among nurses with multiple roles than nurses without (*p* = 0.003). In addition, scores for WFPS and FWPS, both of which are types of positive spillover, were also higher among nurses with multiple roles than nurses without (*p* < 0.001 for both). For UWES-J that assesses work engagement, total and individual scores of the three subscales were higher among nurses with multiple roles (*p* < 0.001 for each).

**Table 2:** Comparisons of variables based on the presence or absence of multiple roles

Variable				All subjects (*N* = 612)			Subjects with multiple roles (*N* = 262)					Subjects without multiple roles (*N* = 350)					*p*
				Median	Range		Median	Range		Percentile		Median	Range		Percentile		
					Min	Max		Min	Max	25	75		Min	Max	25	75	
Personal resource (coping)	BSCP^a^	Active solution for problems	Range 3~12	9.0	3.0	12.0	9.0	3.0	12.0	8.0	11.0	9.0	3.0	12.0	7.0	10.0	< 0.001
		Seeking help for solution	Range 3~12	9.0	3.0	12.0	9.0	3.0	12.0	7.0	10.0	9.0	3.0	12.0	7.0	10.0	0.073
		Changing mood	Range 3~12	8.0	3.0	12.0	8.0	3.0	12.0	6.0	10.0	9.0	3.0	12.0	6.0	10.0	0.002
		Changing a point of view	Range 3~12	7.0	3.0	12.0	7.0	3.0	12.0	6.0	9.0	7.0	3.0	12.0	6.0	8.0	0.206
		Emotional expression involving others	Range 3~12	6.0	3.0	11.0	6.0	3.0	11.0	5.0	7.0	6.0	3.0	11.0	5.0	7.0	0.842
		Avoidance and suppression	Range 3~12	6.0	3.0	12.0	5.0	3.0	12.0	4.0	7.0	6.0	3.0	12.0	5.0	7.0	< 0.001
Work domain	Job demands		Range 1~4	3.0	1.0	4.0	3.0	1.0	4.0	3.0	3.0	3.0	2.0	4.0	3.0	3.0	0.001
	Job resources		Range 1~4	3.0	1.0	4.0	2.0	1.0	3.0	2.0	3.0	3.0	1.0	4.0	2.0	3.0	0.422
Work and family domain (Spillover)	SWING-J^b^	WFNS^c^	Range 0~3	0.9	0.0	3.0	0.9	0.0	3.0	0.5	1.3	0.9	0.0	2.9	0.4	1.1	0.003
		FWNS^d^	Range 0~3	0.0	0.0	2.3	0.0	0.0	2.0	0.0	0.3	0.0	0.0	2.3	0.0	0.5	0.088
		WFPS^e^	Range 0~3	1.0	0.0	3.0	1.2	0.0	3.0	1.0	1.6	1.0	0.0	3.0	0.8	1.4	< 0.001
		FWPS^f^	Range 0~3	1.2	0.0	3.0	1.4	0.0	3.0	1.0	2.0	1.0	0.0	3.0	0.6	1.6	< 0.001
Work Engagement	UWES-J^g^	Total score	Range 0~6	2.6	0.0	6.0	3.2	0.0	6.0	2.0	4.2	2.2	0.0	6.0	1.2	3.3	< 0.001
		Vigor	Range 0~6	2.3	0.0	6.0	3.0	0.0	6.0	1.7	4.3	2.0	0.0	6.0	0.7	3.1	< 0.001
		Dedication	Range 0~6	3.3	0.0	6.0	4.0	0.0	6.0	2.7	5.0	3.0	0.0	6.0	2.0	4.3	< 0.001
		Absorption	Range 0~6	2.0	0.0	6.0	2.3	0.0	6.0	1.0	3.7	1.7	0.0	6.0	0.3	3.0	< 0.001

^a^The Brief Scales for Coping Profile

^b^The Japanese version of the Survey Work–Home Interaction–NijmeGen

^c^Work to family negative spillover

^d^Family to work negative spillover

^e^Work to family positive spillover

^f^Family to work positive spillover

^g^The Japanese version of the Utrecht Work Engagement Scale

*p* comparison of subjects with and without multiple roles using Mann-Whitney *U* test

Simple correlation analysis on the factors associated with work engagement revealed significant positive correlations between the total UWES-J score and age (*r* = 0.194, *p* < 0.01; *r* is Spearman’s rank correlation coefficient), years of experience (*r* = 0.207, *p* < 0.01), BSCP “active solution for problems” (*r* = 0.332, *p* < 0.01), “seeking help for solution” (*r* = 0.217, *p* < 0.01), “changing a point of view” (*r* = 0.272, *p* < 0.01), job resources (*r* = 0.284, *p* < 0.01), WFPS (*r* = 0.306, *p* < 0.01), and FWPS (*r* = 0.438, *p* < 0.01). Negative correlations were obtained with frequency of night shifts per month (*r* = − 0.154, *p* < 0.01), BSCP “avoidance and suppression” (*r* = − 0.172, *p* < 0.01), job demands (*r* = − 0.125, *p* < 0.01), and FWNS (*r* = − 0.083, *p* < 0.05).

Table 3 displays the results of the hierarchical multiple regression analyses conducted. The adjusted *R*^2^ coefficient increased significantly with the addition of variables in each category. The adjusted *R*^2^ coefficient was largest in model 5 (0.324) and factors that were significantly associated with work engagement were FWPS (β = 0.304, *p* < 0.001; β is the standardized partial regression coefficient), job demands (β = 0.181, *p* < 0.001), BSCP “changing a point of view” (β = 0.159, *p* < 0.001), “active solutions for problem” (β = 0.116, *p* < 0.01), presence or absence of multiple roles (β = 0.097, *p* < 0.05), years of experience (β = 0.079, *p* < 0.05), and BSCP “avoidance and suppression” (β = − 0.085, *p* < 0.05).

**Table 3:** Factors associated with work engagement in the hierarchical multiple regression analyses (*N* = 612)

Explanatory variable			Model 1		Model 2		Model 3		Model 4		Model 5	
			β (95% CI)	*p*	β (95% CI)	*p*	β (95% CI)	*p*	β (95% CI)	*p*	β (95% CI)	*p*
Individual factors	Attributes	Years of experience	0.182 (0.010–0.029)	< 0.001	0.122 (0.004–0.022)	0.003	0.135 (0.006–0.023)	0.001	0.101 (0.003–0.019)	0.009	0.079 (0.001–0.017)	0.044
		Frequency of night shifts per month	− 0.067 (− 0.069–0.010)	0.149	− 0.083 (− 0.073–0.001)	0.053	− 0.066 (− 0.065–0.007)	0.119	− 0.044 (− 0.053–0.015)	0.271	− 0.017 (− 0.043–0.028)	0.672
		Type of employment (0, non-regular member of staff; 1, regular member of staff)	0.035 (− 0.183–0.450)	0.409	0.067 (−0.038–0.546)	0.088	0.042 (−0.128–0.448)	0.276	0.042 (− 0.115–0.429)	0.257	0.053 (−0.071–0.475)	0.147
Personal resource (coping)	BSCP^a^	Active solution for problem			0.166 (0.037–0.122)	< 0.001	0.166 (0.038–0.121)	<0.001	0.118 (0.017–0.096)	0.005	0.116 (0.016–0.095)	0.006
		Seeking help for solution			0.036 (− 0.022–0.054)	0.402	0.007 (− 0.034–0.040)	0.874	0.017 (−0.028–0.043)	0.679	0.017 (−0.028–0.043)	0.674
		Changing a point of view			0.242 (0.079–0.155)	< 0.001	0.209 (0.063–0.138)	<0.001	0.162 (0.042–0.114)	<0.001	0.159 (0.041–0.112)	<0.001
		Avoidance and suppression			− 0.197 (− 0.142 – − 0.063)	< 0.001	− 0.170 (− 0.127− 0.049)	<0.001	− 0.093 (− 0.086 − −0.010)	0.014	− 0.085 (− 0.082 − −0.006)	0.025
Work domain	Job demands						− 0.035 (− 0.255–0.089)	0.342	−0.021 (−0.212−0.115)	0.559	−0.021 (−0.213−0.113)	0.546
	Job resources						0.219 (0.277–0.550)	< 0.001	0.176 (0.202−0.463)	<0.001	0.181 (0.212−0.472)	<0.001
Work and family domain (spillover)	SWING-J^b^	FWNS							− 0.010 (−0.188–0.141)	0.780	− 0.010 (−0.189–0.140)	0.770
		FWPS							0.320 (0.352–0.558)	< 0.001	0.304 (0.327–0.535)	< 0.001
Presence or absence of multiple roles (0, without multiple roles; 1, with multiple roles)											0.097 (0.037–0.349)	0.015
Adjusted *R*^2^ coefficient and variation for each model and standardized regression coefficient				0.040		0.190		0.235		0.319		0.324
Adjusted *R*^2^ variation				0.045		0.200		0.247		0.331		0.337
*F* value				9.572		21.520		21.899		26.975		25.425
Significance probability of analysis of variance				< 0.001		< 0.001		< 0.001		< 0.001		< 0.001

## Discussion

The aim of this study was to examine work engagement in female nurses and investigate its relationship with factors such as the presence or absence of multiple personal roles. Results indicated that work engagement of female nurses was low, as is the case with other female workers in Japan. However, the hypothesis that work engagement is higher among female nurses with multiple roles than among those without was supported (H1). In addition, the factors associated with work engagement in female nurses were positive family-to-work spillover, job resources, coping strategies including “changing a point of view,” “active solution for problems,” “avoidance and suppression,” and the presence of multiple roles. Therefore, H2 was also supported partially.

The mean age of subjects in the present study was 35 years, which is roughly the same as the mean age of nurses in Japan [45]. In fact-finding surveys on the employment of nursing staff, the main employment type was being a regular member of staff and most nurses worked in hospital wards [46]. In addition, nurses worked eight or fewer night shifts a month, which is the same as that prescribed in the Guidelines on Night Shift and Shift Work for Nurses Standards for Organizing Shift Schedules set by the Japanese Nursing Association [47]. Therefore, the employment conditions of the subjects in the present study do not appear to differ significantly from the average employment conditions of nurses in Japan.

### Current state of work engagement in female nurses

Based on a previous study, the reference score for the UWES short form was 3.7 points [48], and the mean score of subjects in the present study was 2.6 points. Further, this is roughly the same as the mean score of 2.6 points for women in Japan [6] and 2.6 to 2.8 points for nurses according to previous studies [49–51]. Therefore, it was concluded that the work engagement of subjects in the present study was similarly low as that of female workers and nurses in Japan in general.

The present study showed higher work engagement among female nurses with multiple roles than those without. The female nurses with multiple roles in this study had experienced life events such as marriage, childbirth, and child-rearing, and held roles such as a wife and mother. Previous studies have also found that work engagement is higher among (a) female nurses who are married than those who are not married [25] (b) married female nurses with four or more years’ experience [5], and that nurses with children have a higher sense of achievement regarding work and are less likely to experience burnout, the polar opposite of work engagement [26]. The results of the present study support those of previous studies. Moreover, female nurses with multiple roles were older and more experienced than those without. Previous studies have also found that work engagement increases with increasing age and experience [6, 50] suggesting that the difference in work engagement between the two groups may be influenced by age and years of experience.

### Factors related to the state of work engagement in female nurses

In the hierarchical multiple regression analyses, work engagement of female nurses, which was the dependent variable, was predicted by high FWPS, more job resources, greater use of “changing a point of view” and “active solution for problems” as coping strategies, the presence of multiple roles, and less use of “avoidance and suppression” as a coping strategy, even after controlling for years of experience.

In the present study, based on the JD-R model, we postulated that spillover effects that include variables in both the work and family domains would be associated with higher work engagement, which is an outcome in terms of high WFPS and FWPS, and low WFNS and FWNS. However, for positive spillover, only FWPS, namely, the positive effects of family on work, was associated with work engagement, and there were no associations with any type of negative spillover. A comparison of the spillover effects between the two groups revealed that FWPS was higher among those with multiple roles than those without. This suggests that female nurses with multiple roles may be more influenced by the effects of FWPS, which is a resource that increases work engagement, than by the presence of multiple roles. The circumstances within the family domain that positively affect the work domain may be an important contributing factor. A study on the determinants of work engagement in nurses working in hospitals found that the presence of someone who is pleased by their work contributes to higher work engagement [52]. Female nurses with multiple roles, having family members with whom they share their daily lives and who acknowledge and delight in their work, may build better relationships and increase FWPS, leading to a further increase in work engagement.

Coping strategies that were associated with an increase in work engagement included the greater use of “changing a point of view” and “active solution for problems” strategies, and reduced use of “avoidance and suppression.” These results are consistent with earlier work studying factors related to work engagement of nurses working at hospitals examined by years of experience; this research suggested a greater use of “changing a point of view” and less use of “avoidance and suppression” strategies among nurses with between 4 and 9 years’ experience and 10 or more years’ experience [5]. Another study showed that greater use of problem-focused coping strategies and less use of avoidance coping strategies was related to better work engagement in nurses [53]. Further, according to cross-sectional data on workers, those who often used problem-focused coping strategies or “changing a point of view” as a strategy had stronger stress-mediating factors in the workplace, such as level of control, level of achievement, and support from coworkers. On the other hand, workers who often used an avoidance coping strategy felt more stress from interpersonal relationships and a higher qualitative workload [42]. Moreover, daily rumination has been found to inhibit the use of contextual resources [54]. The current results suggest that having more job resources is related to better work engagement in female nurses, consistent with the JD-R model [3, 4], but that simply having more job resources may not be enough; those resources may not be utilized depending on the types of coping strategies used by the worker. While it is necessary to confront issues and actively seek resolutions to problems, an attitude of avoiding ruminating on one problem for too long and changing perspectives may lead to greater use of job resources, and thus increase work engagement.

Compared to the scores of the standard population calculated in the BSCP development process (9.6 points for “active solution for problems,” 8.0 points for “seeking help for solution,” 7.6 points for “changing mood,” 7.7 points for “changing a point of view,” 4.4 points for “emotional expression involving others,” and 6.4 points for “avoidance and suppression”) [44], the present study showed higher scores among all subjects in “seeking help for solution,” “changing mood,” and “emotional expression involving others,” and lower scores in “active solution for problems,” “changing a point of view,” and “avoidance and suppression,” all of which are related to an increase in work engagement. This suggests that the greater use of “active solution for problems” and “changing a point of view” and the reduced use of “avoidance and suppression” may be important coping strategies as personal resources to enable female nurses to work energetically.

Based on the difference in the presence or absence of multiple roles, nurses with multiple roles had lower scores than the standard population in “changing a point of view” and “avoidance and suppression” and roughly the same scores in “active solution for problems” and “changing mood.” In contrast, scores for “active solution for problems,” “changing a point of view,” and “avoidance and suppression” were lower among nurses without multiple roles compared to the standard population. This indicates that the greater use of “active solution for problems” and “changing a point of view” as coping strategies may increase work engagement in female nurses. In addition, while it has been suggested that the use of both “active solution for problems” and “changing mood” are effective in improving mental health associated with work engagement [55], nurses with multiple roles did not use “changing mood” more often than nurses without multiple roles. This may be explained by the fact that nurses with multiple roles have a spouse and possibly children, unlike nurses without multiple roles, and may be less able to spend time or money on themselves, thus making it difficult to find the time to “change their mood.” [56] Furthermore, nurses with multiple roles did not use “avoidance and suppression” more as compared to those without multiple roles or even compared with the standard population. This may be because nurses with multiple roles cannot avoid responding to the demands of their family and have no choice but to address them in their day-to-day lives. Therefore, it is speculated that one is unable to avoid stressors when coping with them, leading to the realization from daily life experiences that avoiding stressors is not an effective strategy to cope with them. This may lead to a greater tendency in nurses with multiple roles to feel that they have many job resources, and a reduced tendency to feel that there are heavy job demands.

In a similar vein, having greater “job resources” was detected as a determinant of work engagement in the work domain, and this is consistent with the JD-R model [3, 4]. Like previous studies, the current findings suggest that various resources in the work domain may increase positive feelings towards work in female nurses. However, the score for “job resources” of the subjects was lower than that of female employees working in the healthcare and welfare industry in Japan [57]. “Job resources” in the present study refers to resources at the work operation level, department level, and workplace level. Therefore, helping nurses in Japan work energetically may require improving nursing care work as well as the organizational work environment (e.g., for the hospital as a whole and the nurse’s department).

This study has some limitations. First, the participants in this study were limited to those from a single prefecture in Japan, and the response rate was about 50%. Thus, the sample may not be representative of all female nurses in Japan. Although age, years of experience, and employment conditions of the participants in this study are very similar to the average in Japan, further studies with a higher response rate are needed, and collecting more comprehensive data could provide insights into how female nurses can lead a more balanced and active work life.

Second, although we used the results of model 5 that had the largest adjusted *R*^2^ value, it was still only 0.324; therefore, we were able to explain work engagement with predicted variables by only about 30%. The determinants of work engagement used in the present study were presence or absence of multiple roles, coping strategies as a variable related to personal resources, job resources and job demands as variables in the work domain, and spillover effects as a variable in the work and family domain. However, other relevant variables may also influence the level of work engagement [58]. Further research should be conducted to investigate additional variables that may help determine work engagement. Above all, we collected data concerning participants’ evaluation of salaries, which were included in the job resources domain. However, in the future, additional information should be analyzed, including the salaries of nurses’ spouses, household income, and nurses’ satisfaction with both.

Third, the presence or absence of multiple roles was significantly associated with work engagement, but the standardized regression coefficient β was as low as 0.097. This is because multiple roles in this study only include employee, wife, and mother, and caregiver role was not included. Therefore, studies that include caregiver role as part of multiple roles should be conducted in the future.

Lastly, the survey was conducted only with women. Most nurses in Japan are women; however, expanding the scope of research to include men would further elucidate nurses’ work engagement and could help determine the measures that are necessary to improve the quality of nursing care.

## Conclusion

Work engagement of female nurses was low, similar to other female workers in Japan in general; however, work engagement was higher among female nurses with multiple roles than those without. In addition, greater work engagement of female nurses was associated with high FWPS, more job resources, greater use of “changing a point of view” and “active solution for problems” as coping strategies, the presence of multiple roles, and less use of “avoidance and suppression” as a coping strategy. These results indicate that having multiple roles as a result of life events such as marriage, birth, and child-rearing may be one of the positive factors for improving work engagement. Therefore, it is necessary for nurses to receive support at work and use effective coping strategies. This can help reduce female nurses’ perception of difficulties in the balance between work and family.

## Data Availability

All data generated or analyzed during this study are included in this article.

## Abbreviations

BSCP: The Brief Scales for Coping Profile
FWNS: Family-to-work negative spillover
FWPS: Family-to-work positive spillover
JD-R: Job demands-resources
SWING: Survey Work–Home Interaction-NijmeGen
The SWING–J: The Japanese version of the SWING
UWES-J: The Japanese version of Utrecht Work Engagement Scale
WFNS: Work-to-family negative spillover
WFPS: Work-to-family positive spillover
